# Multi-Functionalized Nanomaterials and Nanoparticles for Diagnosis and Treatment of Retinoblastoma

**DOI:** 10.3390/bios11040097

**Published:** 2021-03-26

**Authors:** Rabia Arshad, Mahmood Barani, Abbas Rahdar, Saman Sargazi, Magali Cucchiarini, Sadanand Pandey, Misook Kang

**Affiliations:** 1 Department of Pharmacy, Quaid-I-Azam University, Islamabad 45320, Pakistan; rabia.arshad@bs.qau.edu.pk; 2 Department of Chemistry, ShahidBahonar University of Kerman, Kerman 76169-14111, Iran; mahmoodbarani7@gmail.com; 3 Department of Physics, Faculty of Science, University of Zabol, Zabol 98613-35856, Iran; 4 Cellular and Molecular Research Center, Resistant Tuberculosis Institute, Zahedan University of Medical Sciences, Zahedan 98167-43463, Iran; sgz.biomed@gmail.com; 5 Center of Experimental Orthopaedics, Saarland University Medical Center, 66421 Homburg/Saar, Germany; mmcucchiarini@hotmail.com; 6 Department of Chemistry, College of Natural Science, Yeungnam University, 280 Daehak-Ro, Gyeongsan 38541, Korea; 7 Particulate Matter Research Center, Research Institute of Industrial Science & Technology (RIST), 187-12, Geumho-ro, Gwangyang-si 57801, Korea

**Keywords:** retinoblastoma, rare cancer, surface-tailored multi-functionalized nanoparticles, metallic nanoparticle, tumor-suppressor gene mutation

## Abstract

Retinoblastoma is a rare type of cancer, and its treatment, as well as diagnosis, is challenging, owing to mutations in the tumor-suppressor genes and lack of targeted, efficient, cost-effective therapy, exhibiting a significant need for novel approaches to address these concerns. For this purpose, nanotechnology has revolutionized the field of medicine with versatile potential capabilities for both the diagnosis, as well as the treatment, of retinoblastoma via the targeted and controlled delivery of anticancer drugs via binding to the overexpressed retinoblastoma gene. Nanotechnology has also generated massive advancements in the treatment of retinoblastoma based on the use of surface-tailored multi-functionalized nanocarriers; overexpressed receptor-based nanocarriers ligands (folate, galactose, and hyaluronic acid); lipid-based nanocarriers; and metallic nanocarriers. These nanocarriers seem to benchmark in mitigating a plethora of malignant retinoblastoma via targeted delivery at a specified site, resulting in programmed apoptosis in cancer cells. The effectiveness of these nanoplatforms in diagnosing and treating intraocular cancers such as retinoblastoma has not been properly discussed, despite the increasing significance of nanomedicine in cancer management. This article reviewed the recent milestones and future development areas in the field of intraocular drug delivery and diagnostic platforms focused on nanotechnology.

## 1. Introduction

Retinoblastoma (RB) is an aggressive ophthalmological cancer found during childhood and infancy but an uncommon malignancy of older children and young adults, with a worldwide prevalence of 1/15,000 to 1/20,000 live births [1]. Strabismus and leukocoria are two major signs of RB [2]. If not treated in the early stages, it often causes devastating consequences, such as the loss of vision, secondary nonocular tumors, and even death [3,4]. As a highly malignant tumor, RB usually manifests in the first three years of life and represents the prototypic pattern for inherited tumors, with the tumor initiated by the somatic inactivation of both alleles of the RB gene (*RB1*) [5,6]. RB tumors, deriving from the immature cells of the retina, have a large amount of heterogeneous vasculature and depend on the vascular supply for their proliferation [7].

The traditional treatments of children with RB used to be external beam radiotherapy, episcleral plaque radiotherapy, enucleation, cryotherapy, and photocoagulation [8]. Over the last decade, RB treatment has changed enormously. In this respect, with changing attitudes towards concerns over radiotherapy, intravenous and intra-arterial chemotherapies became the cornerstone of RB treatment, since they have been shown to effectively decrease the tumor’s size, prevent the spread of the disease, and preserve vision [9]. Yet, their clinical application is limited due to possible systematic toxicity, drug resistance, and rapid blood clearance [10]. New therapeutic strategies, which are more intensive and demand an integrative approach, were put into practice to avoid deleterious complications of the aforementioned modalities, including cataracts, radiation retinopathies, and facial deformities [3,11].

The conventional administration of chemotherapeutic drugs decreases their clinical efficacy, particularly for water-insoluble drugs and for delivering such medications to the eye’s posterior segment [12,13,14,15,16,17,18]. In order to overcome this therapeutic obstacle, the local delivery of these drugs—explicitly, alkylating agents—to the eye has gained much attention as a beneficial strategy for minimizing systematic complications, such as ischemic necrosis, orbital fat necrosis, atrophy of the optic nerve, and changes in ocular motility [19,20]. These unfavorable effects are probably caused by prompt dispersion of the aqueous solution of alkylating drugs to the orbital, periorbital, and surrounding tissues [21]. However, the delivery of eye drugs continues to pose a serious challenge due to the clearance of conjunctival vessels. Besides, the existence of physiological and anatomical barriers of the eye, comprised of ocular surface epithelium, blood–retina barriers, and blood–aqueous barriers, might limit its efficiency [12]. From this perspective, the establishment of productive delivery transporters is therefore essential for RB therapy.

In terms of the diagnosis, clinicians routinely confirm RB by the appearance of retinal tumors using needle biopsy, fluorescein angiography, commutated tomography and ultrasonography, and magnetic resonance imaging (MRI) [6]. Despite the availability of these ophthalmic imaging modalities, there is still an urgent need, particularly for ocular molecular imaging, that further enables the early detection of eye disorders prior to the appearance of grossly visible morphological alterations [22]. In those cases, ophthalmic tumors are typically diagnosed when they can no longer be regarded as malignant cells but malignant tissue. On the other hand, biopsy sampling can provide us with invaluable information concerning the histological type of the ophthalmic tumor, and sampling errors might cause falsely negative specimens [23].

Recently, the use of nanotechnologies has been experiencing exponential growth in the diagnosis and treatment of tumors and eye disorders. Nanoparticles (NPs), nanocages, nanocapsules, nanoliposomes, nanohydrogels, nanodendrimers, and nanomicelles are amongst the most applicable nanotechnology-based ocular delivery systems providing several advantages over routine diagnostics/therapies [24,25,26,27,28,29,30]. Owing to their unique characteristics and potential applications in medicine and biology, nanomaterials (NMs) were developed to revolutionize disease diagnoses, treatments, and therapies [31,32,33,34,35,36]. Currently, NPs are extensively used for the effective delivery of drugs, small molecules, peptides, nucleic acids, and even vaccines [37]. Thanks to their controlled release, nanometer-scale dimensions, and desirable therapeutic toxicity, NPs yield promising outcomes even at very low concentrations and have fewer side effects than traditional chemotherapeutics [38,39]. This makes NPs an object of even broader interest for being used in implants, NP-contained contact lenses, films, nanofabricated devices, and designed nanocarriers for ocular drug delivery [39].

Different NPs can be administrated into the eye by different routes, including topical, periocular, systemic, intravitreal, and suprachoroidal. In this regard, suprachoroidal, intravitreal, and periocular administration is recommended for either slow-release NPs or stimuli-responsive NPs. Simultaneously, bioadhesive or rapid uptake NPs are better for being topically injected [40]. Functionalizing NPs with peptides and protein ligands, such as transferrin, could facilitate their conjunctival entry and transport [41]. Another example is applying magnetic NPs loaded with a drug payload to enhance the cellular uptake of the payload [42]. These functionalized NPs can be utilized in ocular delivery systems and serve as novel promising contrast agents for MRIs [40]. Furthermore, NPs provide many advantages for delivering nucleic acids (i.e., RNA, short interfering RNS, and microRNAs) [43]. The advantages of gene transfection using NPs include (i) enhancement of the cell entry of nucleic acids, (ii) protection of nucleic acids from degradation by nucleases in the body and increasing the duration of gene transfection, (iii) avoiding nucleic acids to bind to specific cell surface receptors and reduce their off-target effects [44,45]. Therefore, functionalized NPs could be regarded as desirable tools for retinal gene therapy and nucleic acid delivery for treating other ophthalmic pathologies—specifically, RB [46,47].

Nanoparticle drug delivery is a novel approach to the treatment of eye disorders. Still, there are several challenges in using NPs for such purposes [48,49,50,51,52,53,54,55,56]. For one thing, as a drug or gene carrier, NPs do not affect the retina and cornea [12]. Moreover, for using some NPs, long incubation periods are needed [57], and NPs might release insoluble particles and factors that interact with biological systems [58]. In this review, we discuss the application of NPs in RB and explore the future perspectives of NPs as aids in the diagnosis and treatment of RB.

## 2. The Role of Nanotechnology in the Diagnosis of Retinoblastoma

The risk of intraocular cancer problems and metastatic potential needs to be controlled in the same way as other cancers [9]. The early detection of intraocular cancer is important for maintaining vision because of its proximity to vital ocular tissues [59]. There are two types of intraocular tumors that can be classified according to the average age of incidence: childhood RB and adult ocular melanoma [60].

In children under five years of age, RB, often an inherited disease, occurs and is induced by down regulation of the RB gene. In developing countries, the prevalence of this type of intraocular cancer is higher. The silencing of this gene eliminates the cell cycle regulation restrictions that lead to the uncontrolled proliferation of cells [61,62]. Ocular inflammation due to extra ocular colonization of the tumor can be seen in severe instances. RB can spread into the pleural cavity and to the brain and spinal cord. The choroid vasculature may also be infiltrated and distributed to the bone and stem cells [1].

An RB diagnosis is usually performed by an ophthalmologist’s evaluation and imaging of the eye. Fundoscopy identification usually reveals a large white-to-creamy-colored tumor with retinal and vitreous space lesions. Ultrasonography is used to classify and evaluate intraocular tumors, because CT scans are not advised for young kids [63,64,65]. Additionally, the extra ocular extension of the tumor is studied through magnetic resonance imaging (MRI) of the brain and the orbits [66]. Traditional optical imaging and ultrasound imaging, considering the flexibility of the ophthalmic imaging methods, are not effective at identifying the early pathologies of eye diseases until morphological changes are apparent.

On the other hand, in order to improve health outcomes, several diagnostic methods have been developed. However, when using the existing techniques, there are restrictions and limitations [67]. Additionally, several studies have recently established new RB biomarkers that can be used as prognostic factors for diagnosis and can contribute to the understanding of RB pathogenesis and help address potential treatments and diagnosis approaches [68].

The early detection of RB is a key feature in successful treatment [69]. Nanotechnology offers new molecular contrasting agents and nanomaterials for earlier and more reliable initial detection and continuous monitoring of the treatment of cancer patients [15,16,25,27,70]. Recently, several nanoplatforms have been developed to enhance the image quality of the traditional imaging techniques (See Table 1) [49,50,54,71]. Amidst this progress, inadequate studies have been conducted to enhance the performance of traditional ocular imaging techniques such as MRI, ultrasound imaging, and optical coherence tomography through the use of nanoplatforms [72]. Despite inadequate research efforts in this area, these nanoplatforms have demonstrated tremendous potential for enhancing the imaging and diagnosis quality of retinal diseases.

Quantum dots (QDs) have been evaluated for their applications in ocular imagery. They have excellent optical durability and can make multimodal detection easier [80,81]. The injection of cultured human corneal endothelial cells (cHCECs) into the anterior chamber is a newly developed modality for the successful treatment of corneal endothelium dysfunction. For instance, to monitor injected cHCECs, Toda et al. investigated cultured human corneal endothelial cells (cHCECs) labeled by semiconductor QDs. They explored the efficacy of in vivo fluorescence imaging in a corneal endothelial dysfunction mouse model to study the dynamics and aggregation of QD-labeled injected cHCECs [77]. In this study, no morphological alteration in the cHCECs or the expression of functional markers of cHCECs were induced by QD-labeling. The injected cHCECs QDs were quantified. The retention of cHCECs QDs was obvious in the cryogenically injured corneal endothelium mouse model eyes from 3 to 48 h post-cell injection on the posterior surface but not in the non-injured healthy control eyes. QDs may be good contrast agents if the toxicity of the dots is considered. Some researchers have proposed AuNPs as an alternative.

AuNPs can act as perfect contrast agents for imaging, in addition to QDs, and several authors have used AuNPs for imaging eye cancers over the past few years [82]. For example, Cruz-Alonso et al. described an immunohistochemical approach for visualizing the distribution of metallothionein 3 (MT3) and metallothionein 1/2 (MT 1/2) in human ocular tissue [74]. In this methodology, Au nanocluster (AuNC)-connected antibodies are used as markers and can be coupled with ICP-MS. Water-soluble fluorescent AuNCs with an average size of 2.7 nm were prepared by carbodiimide coupling and then covalently linked to antibodies. To prevent nonspecific contact with biological tissue, the surfaces of the modified AuNCs were then blocked with hydroxylamine. The signal enhancement by >500 Au atoms in each nanocluster enabled LA-ICP-MS to identify the antigens (MT 1/2 and MT 3) using a laser spot size as small as 4 μm. In this study, the picture patterns found in the retina were in good agreement with those obtained by the traditional immunohistochemistry of fluorescence.

In another study, for the first time, Lapierre-Landry et al. examined in vivo photothermal optical coherence tomography (PT-OCT) in the eye for endogenous (melanin) and exogenous (Au nanorods) absorbers [75]. In retinal imaging, OCT has become a quality of treatment. OCT facilitates noninvasive tissue architecture mapping but lacks the specificity of contrasting agents that could be used for in vivo molecular imaging. PT-OCT is a practical technique based on OCT that was produced in a sample to identify the absorbers. To separate the photothermal signal from melanin in the retina, pigmented mice and albino mice were used. After the systemic injection of Au nanorods to investigate their passive aggregation in the retina, pigmented mice with laser-induced choroidal neovascularization lesions were also visualized. The current research has demonstrated the capacity of combining the PT-OCT method with Au nanorods to image the distribution of both endogenous and exogenous absorbers in mouse eyes.

In another study, Kim et al. showed the medicinal use of fucoidan-coated Au NPs and those encapsulated by doxorubicin (DOX) for the in vivo and in vitro dual photothermal therapy (PTT) and chemotherapy of eye tumors [73]. Marine-derived fucoidan was used to obtain a higher photostability for AuNPs as a capping agent, and DOX was loaded to stimulate a chemotherapy anticancer drug. The prepared DOX-fucoidan@AuNPs demonstrated high tumor cell cytotoxicity and good light absorption for in vitro temperature rises. Following an intratumoral injection of DOX-fucoidan@AuNPs into rabbit eye tumors, PTT-assisted NPs resulted in the complete and nonrecurrent elimination of eye tumors for 14 days after the procedure. Due to responsive light absorption by the administered NPs, the photoacoustic image contrast from the tumor tissues was improved dramatically. Interestingly, the use of marine-derived fucoidan, along with AuNPs, can improve the ability of AuNPs for better photothermal therapy.

Altundal et al. also explored the dosimetric possibility of using AuNPs or carboplatin-loaded AuNPs to increase the effectiveness of radiotherapy for ocular cancers (choroidal melanoma) and RB during kV energy external and internal beam radiotherapy [83]. The data predicted that using AuNPs or carboplatin-loaded AuNPs combined with radiation therapy for ocular cancer utilizing kV energy photon beams could achieve major dose improvements. In the kV energy range, brachytherapy sources produce higher dose improvements than an external beam. The external beam, however, has the benefit of being noninvasive.

The effectiveness of brachytherapy with ultrasonic hyperthermia modality in the existence of AuNPs on ocular RB tumors was tested by Moradi et al. in arabbit model [84]. The tumor area was assessed at day zero and the end of the third week using a B-mode ultrasound imaging approach. For a histopathological analysis of the tumor necrosis, the groups were investigated. A high difference between the relative tumor area changes in the combination group and the other study groups was observed. The necrosis of living RB cells was supported by the findings of the histological examination. Once again, Au NPs demonstrated a high ability in different imaging techniques, such as ultrasounds. Therefore, AuNPs can be a better alternative to quantum dots.

Due to their structural differences and their wide range of functionally based electrical and chemical characteristics, carbon nanomaterials have received much interest these days [85]. Researchers have concentrated on electroanalysis using carbon materials for biomolecules, because electrochemical techniques give the benefits of flexibility and responsiveness in constructing a sensor design [86]. Goto et al., for example, described the direct electrochemical identification of DNA methylation using a nanocarbon film electrode in relatively long sequences. The film was developed using the sputtering method of electron cyclotron resonance and had a mixed bond structure of nanocrystalline sp(2) and sp(3) [78]. Their strategy of methylation identification calculated the variations between both the 5-methylcytosine and cytosine oxidation currents without a bisulfite reaction or labeling. The film electrode enabled the quantitative identification of DNA methylation ratios under optimized conditions and sensor measure methylated 5’-cytosine-phosphoguanosine (CpG) repetition oligonucleotides (60 mers) with different methylation ratios. Despite the high ability and low toxicity of carbon nanostructures, there are a few papers for the diagnosis of RB.

In an in vitro setting, magnetic NPs that can provide great contrast for MRIs have, so far, been effective [87]. Previous studies have shown that human serum albumin-coated iron oxide (IO) NPs (HSA and IO/HSA NPs) increase the half-lives of cross-linked therapeutic factors, implying that they can be used for the controlled delivery of therapeutics [88]. To follow other applications, Tzameret et al. evaluated the in vivo monitoring by MRI and the long-term protection of IO/HSA NP delivery into the suprachoroid of a rat retinalmodel [88]. Jaidev et al. synthesized NPs of fluorescent iron oxide in another study and tested their effectiveness against RB cell imaging [76]. Using oleic acid, the iron oxide NPs were prepared and stabilized. Sulforhodamine B was adsorbed onto albumin over NPs of oleic acid-capped iron oxide. In MRI studies, the nanomaterials exhibit a great negative contrast to natural, as well as cancer, cells without cytotoxicity, suggesting their bioavailability. Until now, iron oxide (IO) NPs have been the most used NPs in MRIs. The coating process can decrease some of the stability and toxicity issues.

The combination of nanotechnological strategies in cancer imaging makes it important for their application in ocular diagnostics. Multi-functional nanostructures enable the intraocular tumor responses to different localized chemotherapeutic drugs to be monitored simultaneously in the eye. In this light, a multi-functional nanostructure for multimodal low-intensity centered ultrasound (LIFU)/immune synergistic RB therapy driven by imagery was reported by Wang et al. In order to encapsulate perfluoropentane (PFP) and muramyl dipeptide (MDP), magnetic hollow mesoporous Au nanocages (AuNCs) connected to Fe_3_O_4_ NPs(AuNCs-Fe_3_O_4_) were prepared. The multi-functional magnetic NPs improved the in vivo and in vitro photoacoustic, ultrasound, and magnetic resonance imaging, which was effective for the treatment and efficacy imaging. Upon accumulation in tumors via a magnetic field, the NPs underwent phase transition under LIFU irradiation, and MDP was released. AuNCs-Fe_3_O_4_/MDP/PFP strengthened LIFU’s therapeutic effect and led to direct tumor apoptosis/necrosis, while MDP facilitated dendritic cell (DC) maturation and activation and allowed DCs to recognize and clear tumor cells. The multi-functional AuNC-Fe_3_O_4_/MDP/PFP NPs showed great potential for multimodal imaging-guided LIFU/immune synergistic therapy of RB by improving photoacoustic, ultrasound, and magnetic resonance imaging and inhibiting tumor development.

In the efficient diagnosis of RB, we believe that the combination of different NPs with different abilities can be the best approach.

## 3. Nanoparticles in Treatment of RB

**Figure 1:**
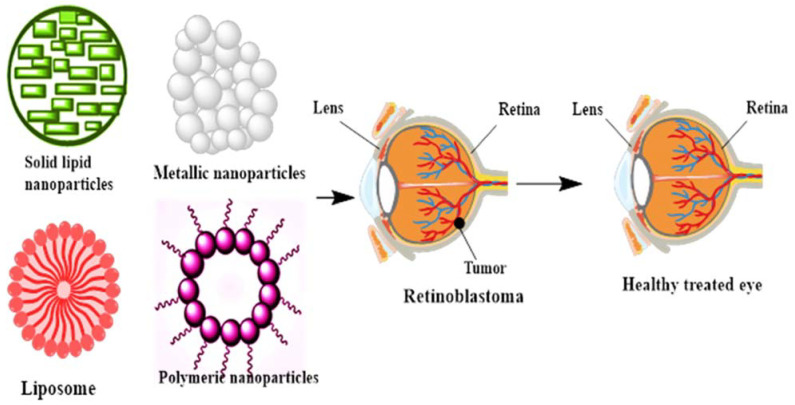
Multi-functionalized nanomaterials for ocular drug delivery to overcome the ophthalmic barriers and treat retinoblastoma.

As mentioned earlier, RB is caused by mutations in the tumor-suppressor gene RB1 and is the most common pediatric eye cancer [89]. RB’s survival rate has decreased in developing countries, and a delayed diagnosis is due to a lower socioeconomic status [90]. Suppressor gene mutations lead to the activation of proliferation and malignancy [91]. The treatment of RB is limited to enucleation [92]. External beam radiation therapy is also in progress, but all chemotherapeutic and radiation therapy is linked with neutropenia, thrombocytopenia, renal toxicity, systemic toxicity, and hepatotoxicity [93,94,95,96]. Therefore, drug delivery in the eye is challenging, owing to defensive barriers in the ophthalmic tissues. Moreover, drug delivery by various nanoformulations is proficient enough to overcome these limitations [97,98]. The most common and useful multi-functionalized NPs in treating RB are multi-functionalized NPs and lipid-based NPs, as well as metallic NPs [99,100]. However, the multi-functionalized nanomaterials for ocular drug delivery to overcome ophthalmic barriers and treat RB is shown in Figure 1**.** However, synthesized NPs have the capability of encapsulating the therapeutic moiety and increasing the retention time [101]. Polymeric NPs are biodegradable in nature and have the capability of intravitreal delivery in RB with specificity and safety. The key features of the nanocarriers for the treatment of RB are listed in Table 2.

## 4. Multi-Functionalized Nanocarrier Therapies for Targeting RB

**Figure 2:**
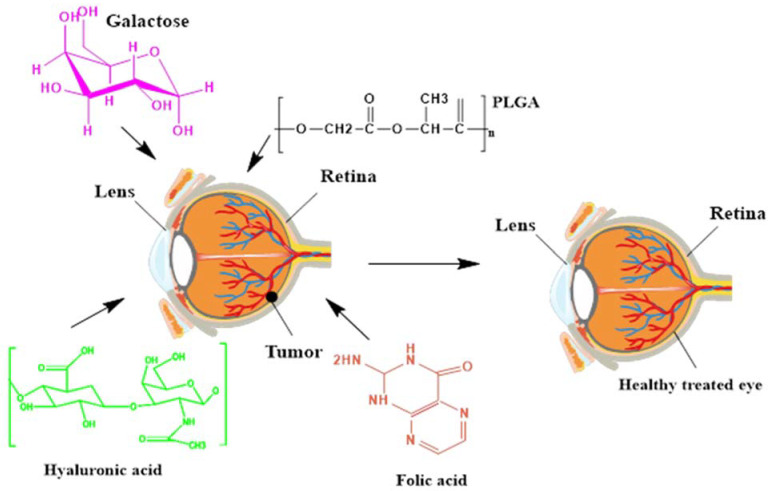
Multi-functionalized ligand-based nanoparticles for targeting retinoblastoma. PLGA: poly-d,l-lactic-co-glycolic acid.

Multi-functionalized NPs are synthesized for the purpose of targeted action via the attachment of specified ligands to target the tissues that are highly overexpressed in a certain disease. These NPs proved to be a benchmark in a plethora of infectious diseases, as well as malignant cancers. In infectious diseases, several ligands are attached to make the nanocarriers system multi-functional for targeting intracellular pathogens. However, in cancers—especially, RB—various biomarkers are overexpressed, i.e., folic acid, hyaluronic acid, and galactose, as shown in Figure 2. Multi-functionalized NPs are synthesized to attach such overexpressed ligands for targeting RB cells, and proficient anticancer activity has been observed [109,110].

### 4.1. Surface-Modified Melphalan Nanoparticles for the Intravitreal Chemotherapy of RB

Lee B. Sims et al. [102] synthesized a two-step formulation via the sing and double-step emulsion technique. Poly-d,l-lactic-co-glycolic acid (PLGA) NPs were synthesized in the first step using the emulsion solvent evaporation technique by encapsulating the fluorescent dye coumarin 6 (C6) for enabling visualization through fluorescence microscopy. Coumarin encapsulation was developed via formulating an oil-in-water (o/w)-based single emulsion technique. Batches (100–200 mg) of the emulsion were synthesized using carboxyl-terminated PLGA. Afterward, C6 was instilled in dichloromethane (DCM) overnight with a final addition of a small quantity of PLGA. The final formulated PLGA/C6/DCM was added to a 5% polyvinyl alcohol (PVA) solution followed by vortexing and sonication, as well as three hours of solvent evaporation. The second step of the formulation involved the incorporation of the drug into melphalan PLGA NPs through the double-emulsion method. The double-emulsion method was utilized to reduce melphalan spilling during the fabrication process. For the development of the double-emulsion technique, PLGA was dissolved in DCM overnight, and melphalan was dissolved in the EDTA buffer. Afterward, melphalan/EDTA was dissolved in the preformed PLGA/DCM mixture, followed by continuous stirring. The resultant mixture was dropwise added into the 5% PVA solution, and the final combined conjugate consisting of PLGA/DCM/melphalan/PVA solution was vortexed, and sonication was performed. The resulting final nanoparticles were hardened for the prevention of melphalan spilling from NPs during synthesis.

### 4.2. Galactose Functionalized Nanocarriers

The sugar moieties ligand-based mechanistic approach for improved and targeted therapy against RB is highly in demand. In RB, sugar moieties in the form of lectins are highly overexpressed as compared to healthy cells. Therefore, overexpressed lectins are a means of targeting for achieving efficacious results. In this research, a novel sugar receptor-targeted delivery system for the safe and targeted delivery of etoposide (ETP) was developed by Godse et al. via conjugating a galactose carboxyl group with amino groups of chitosan (GC) via following carbodiimide chemistry [103]. In the first step of the synthesis, ETP was loaded into poly (lactide-coglycolide) PLGA NPs using the displacement method. The formulated ETP-PLGA NPs were further coated with galactose conjugate, followed by overnight incubation at room temperature with continuous stirring. NPs were separated using ultracentrifugation at 34,000× *g* for 20 min, and the obtained pellet was washed and resuspended in distilled water. The synthesized NPs were characterized by Fourier-transform infrared spectroscopy (FTIR), NMR, entrapment efficiency, size, zeta (*ζ*)-potential, polydispersity index (PDI), in-vitro drug release, and uptake studies. The results concluded the size of NPs in the range of 150–160 nm with a positive ζ-potential and sustained drug release for 32 h. Moreover, the entrapment efficiency of the NPs was about 70%. NPs uptake studies confirmed that chitosan (GC)-conjugated ETP loaded poly (lactide-co-glycolide) (PLGA) nanoparticles (NPs), i.e., ENP was 70% higher than nonconjugated NPs, indicating a targeted delivery against RB [103].

### 4.3. Hyaluronic Acid (HA) Functionalized Nanocarriers

Hyaluronic acid is a FDA-approved marine polymer with great flexibility, biodegradability, shielding, and mobility, as well as anticancer activity, owing to the receptor of CD44 [104]. In 2015, Martens et al. developed a unique treatment option for retinoblastoma in the form of retinal gene therapy [111]. In this research, nonviral polymeric gene DNA complex-based nanomedicines were coated electrostatically with HA for providing an anionic hydrophilic coating for improved intravitreal mobility. The authors further evaluated the resulted polyplexes with HA of different molecular weights by means of size, surface charges, zeta potential, and complexation. It was observed that the *ζ*-potential were four-fold more anionic in the presence of more HA concentrations as compared to low concentrations of HA. It was concluded from the results after developing an ex-vivo model based on excised bovine eyes and fluorescent single-particle tracking (FSPT) that HA-coated polyplexes had improved mobility in intact vitreous humor, as well as proficient uptake through HA-based CD44-receptor endocytosis [111].

### 4.4. Folic Acid (FA) Functionalized Nanocarriers

Nanocarriers can become more effective in the targeted killing of cancerous cells as compared to systemic chemotherapy after they are coupled with targeting moiety [112]. Targeted moieties enable the site-specific delivery of anticancer drugs [113]. The most utilized targeting moiety in practice is the folate receptor. Folate receptors are overexpressed in RB cells, and their use in RB treatment will be highly effective in the preferential uptake of NPs, as well as killing of only cancer cells [105]. Parveen and Sahoo synthesized chitosan NPs (CNPs) and loaded DOX in it [3]. Prepared NPs were conjugated with folic acid. Chitosan nanoparticles (CNPs) can be conjugated to DOX via the ionic gelation method, followed by centrifugation at 18,000 rpm for 30 min at 4 °C for collecting CNP pellets. Afterward, CNPs were lyophilized and stored at 4 °C and coupled with folic acid with a coupling reaction by mixing in distilled water and centrifugation at 3000 rpm.

Moreover, the conjugation of folic acid (FA) onto CNPs was characterized via nuclear magnetic resonance (NMR) and Fourier-transform infrared spectroscopy (FTIR). The cytotoxic effects of synthesized conjugated NPs were assessed on RB cells (Y-79) using the 3-(4,5-dimethylthiazol-2-yl)-2,5-diphenyltetrazolium bromide (MTT) assay (a colorimetric assay for assessing cell metabolic activity), and excellent cytotoxic effects towards RB cells as compared to unconjugated DOX-CNPs and pure DOX. Furthermore, the mechanism of DOX-mediated apoptosis in Y-79 cells was evaluated, and the results concluded that the FA-DOX-CNPs activated the mitochondrial pathways and triggered the release of cytochrome c and caspases enzymes for further assistance in apoptosis. Therefore, FA-targeted NPs were concluded to be sustained, effective, and targeted therapy against RB. Moreover, de MoraesProfirio and Pessine (2018) synthesized FA-conjugated chitosan-coated PLGA NPs for the safe and targeted delivery of carboplatin by using a 2^2^ factorial design and optimized the formulation and characterized by all the optimum characterization techniques needed [114]. The results concluded the NP size of 178 nm, PDI = 0.20, *ζ*-potential = 46.0 mV, encapsulation efficiency = 35.5%, and NP yield = 92%. In the treatment of RB via multi-functionalized NPs, as discussed above, researchers developed sugar moieties-based ligands and polymer-based ligands. Sugar moieties were in the form of lectins. Galactose and HA are highly overexpressed in RB as compared to healthy cells. Therefore, overexpressed lectins are a means of proficient targeting as compared to the other ligands. However, polymeric ligands like PLGA resulted in the highest stability. In our opinion, if polymeric, as well as sugar, moieties are combined and functionalized in the nanosystem, then excellent treatment targets will be achieved with high specificity and stability.

## 5. Lipid Nanoparticles (LNPs)

Lipid nanoparticles (LNPs) are valuable aspects of nanotechnology, being utilized in pharmaceutics and nutraceuticals, as well as cosmetics. Most lipid-based bioactive compounds, i.e., fatty acids, flavonoids, tocopherols, polyphenols, carotenoids, and preservatives, possess a hydrophobic nature [115]. The encapsulation of all these mentioned lipids in the form of colloidal dispersions in the aqueous environment of the oil-in-water (o/w) type is an utmost requirement to ensure the stability of the formulations [116]. LNPs have gained much importance in treating cancers and infectious diseases, as well as the adsorption of heavy metals. Melphalan is the drug of choice as a chemotherapeutic agent for treating RB. However, the risk of immunogenicity and devastating healthy cells is unavoidable [117]. To overcome the disadvantages and to ensure ideal delivery and treatment, Tabatabaei et al. (2019) developed 171-nm switchable LNPs for the codelivery of melphalan and miR-181 with 93% encapsulation efficiency [106]. To prepare melphalan-loaded LNPs (LNP/melphalan), a melphalan and ethanol mixture was added into the lipid mixture to form 10% of the total lipids. Ethanol was then evaporated to form a thin lipid film and again hydrated with 5% dextrose in water for 30 min at 40 °C followed by incubation to develop the LNPs. Next, melphalan was quantified, and the encapsulation of miR-181a was executed. The encapsulation efficiency of miR-181 was determined indirectly via the fluorescence displacement assay. Various characterization techniques have been utilized to assess formulated NPs, and the results showed that LNPs increased the expression of apoptotic genes and the highest uptake and targeted killing of RB cells [106].

### 5.1. Solid Lipid Nanoparticles (SLNs)

SLNs are versatile lipid-based nanocarriers systems enriched with the synergistic qualities of polymeric particles, liposomes, and emulsions. SLNs are synthesized from solid lipid blends via containing lipid droplets that are crystalline in a highly ordered structure and composed of bioactive compounds in the lipid matrix part. The bioactive compounds mobility can be controlled via controlling the physical state of the SLN lipid matrix. The advantages related to SLNs include controlled drug release, drug targeting, encapsulation efficiency, and drug stability [118,119,120]. Ahmad et al. (2019) synthesized SLNs for the safe and targeted delivery of etoposide against RB [121]. SLNs were synthesized via the techniques of melt-emulsification and ultrasonication. Optimization of the novel SLNs was done through a three-factor levels Box-Behnken design for establishing the functional relationship between the response variables of the particle size, surface morphology, and entrapment efficiency (EE). Moreover, the SLNs were characterized for size, surface morphology, entrapment efficiency, and in-vitro drug release. However, pharmacokinetic studies were carried out after the intravitreal administration of SLN formulation in Wister rats. Furthermore, a gamma scintigraphic analysis was performed to check the deposition of SLNs in the ocular tissues of albino rabbits. Gamma scintigraphy involves the injection of radioisotopes (called radiopharmaceuticals) into the bloodstream that actively seek out bone that is irritated or destroyed or rebuilt or tissues that are inflamed or necrotic. Later on, histological studies were performed to assess the toxicity and morphological changes after treatment. However, it was concluded from the results that the particle size, PDI, and EE of the optimized formulation were 239.43 nm, 0.261 ± 0.001, and 80.96% ± 2.21%, respectively. The most advantageous aspect of this formulation was its sustained drug release for seven days with only a single intravitreal administration. The sustained drug release for seven days was also confirmed and supported by the results of the gamma scintigraphy study. The histological studies confirmed the nontoxic nature of the SLNs, as the posterior tissues of eyes did not exhibit detrimental effects. Therefore, it can be obvious that etoposide-loaded SLNs are efficacious and safe in treating RB [121].

### 5.2. Nanoliposomes

**Figure 3:**
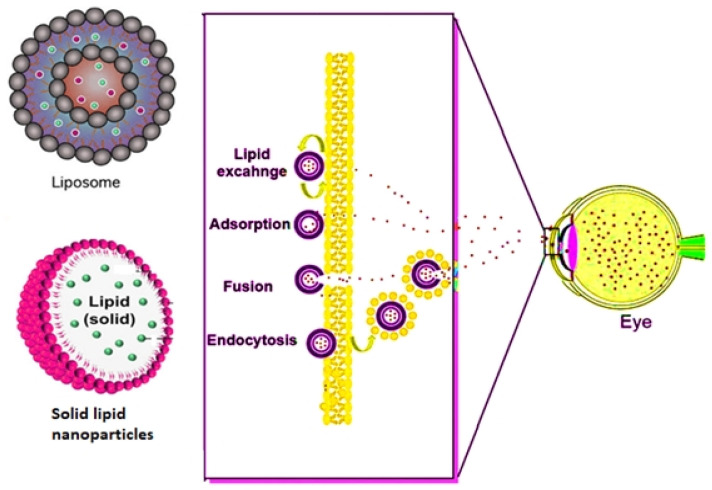
Mechanism followed by lipid and polymeric nanocarriers in overcoming the ophthalmic barrier.

Lipids, when placed in contact with water, the hydrophobic system of the molecule interacts with water, leading to the self-assembly of lipids via forming liposomes (Figure 3) [50]. Liposomes consist of an aqueous core encapsulated by a lipid bilayer and often functionalize via ligand attachments [122,123]. Zhao et al. (2020) synthesized cisplatin nanoliposomes to determine the apoptosis regarding the RB cell lines in vitro, as well as in vivo [124]. Y-79 cells were cultured, and their exposure with Annexin V/propidium iodide (PI) was tested for determining apoptosis. In order to detected cell death, Annexin V/PI double staining kit are used in flow cytofluorimetric analyses. The Annexin V corresponding signal provides a very sensitive method for detecting cellular apoptosis, while propidium iodide (PI) is used to detect necrotic or late apoptotic cells, characterized by the loss of the integrity of the plasma and nuclear membranes. Y-79 cells were also evaluated for the determination of caspase-3 in order to assess any change in inflammatory caspase-3 as well, as it was also determined based on Western blotting for testing various expressions of Bcl-2 and Bax expression proteins. The Y-79-transplanted tumor model in nude mice was done and divided into three groups (*n* = 5). The control group of nude mice was injected with cisplatin, and the blank group of mice was administered with saline. After injecting, the nude mice were slaughtered, and the tumors were removed. After removing of the tumors, the total volumes and weights of the tumors were compared. Furthermore, nucleic acid extraction was done with magnetic beads for the extraction of DNA and RT-PCR, and an in-situ cell death assay kit was applied in testing the apoptotic cells. Furthermore, after comparing the reduction rate of the tumors, the cisplatin liposome group showed a higher Y-79 apoptotic rate, caspase-3, lower volume and weight of the tumors, and Bax protein expression as compared to the cisplatin solution and dimethyl sulfoxide (DMSO) groups, with a significance of *p* < 0.05 [124].

In this research, researchers utilized LNPs to treat RB, and they discovered that LNPs are the most promising for encapsulating hydrophobic drugs via improving the oral bioavailability. Various LNP (SLNs, liposomes, and core-shell nanostructures)-based techniques have been utilized in the past for the successful loading of anticancer drugs and resulted in several limitations of limited drug loading, instability, high cost, poor industrial scaling, and the use of organic solvents. In our opinion, to mitigate the side effects of various lipid formulations, self-emulsifying carriers should be introduced in the targeted killing of RB. Self-emulsifying drug delivery systems (SEDDS) have drawn innumerable attention in the field of pharmaceutical technology and drug development owing to their thermodynamic and kinetic stability and easy manufacturing, as well as a distinct feature of solubilizing both hydrophilic and hydrophobic drugs.

## 6. Metallic Nanoparticles

The treatment of cancer is challenging due to the nonspecific distribution of chemotherapeutic agents in the whole body, causing systemic toxicity and poor patient compliance. Metallic NPs have a great importance in the field of cancer treatment. Similarly, RB, a rare type of cancer, can be treated effectively via the application of metallic NPs following either active or passive targeting [49,125,126].

### 6.1. Silver Nanoparticles (AgNPs)

AgNPs are widely utilized in cancer therapeutics because of their green synthesis, cost-effectiveness, stability, and optical properties [107,127]. Remya et al. reported the synthesis of AgNPs via rapid methodology from natural sources of brown seaweed *Turbinaria ornate*, and its cytotoxic efficacy was determined against RB cells [128]. The synthesis of AgNPs was confirmed via UV-visible spectroscopy and was further characterized by X-ray diffraction (XRD), high-resolution transmission electron microscopy (HR-TEM), ζ potential, potential, thermogravimetric analysis (TGA), and Fourier-transform infrared spectrum (FTIR), as well as advanced plasma mass spectroscopic techniques. The total phenolic content of synthesized AgNPs was found to be 43 nm, with good scavenging activity. Moreover, the cytotoxicity of the synthesized AgNPs against the RB Y-79 cell line showed a dose-dependent response via the inhibitory concentration (IC_50_) of 10.5 µg/mL. The results concluded that AgNPs are promising anticancer agents with enhanced ocular targeting and treatment [128]. Same group researchers (2018) introduced the polysaccharide laminarin into their preformed Ag-NPs and extracted, purified, and analyzed laminarin through Matrix-Assisted Laser Desorption Ionization Time-of-Flight Mass Spectroscopy (MALDI-TOF MS) and Proton Nuclear Magnetic Resonance (^1^H NMR), UV-vis, FTIR, XRD, and TEM. Moreover, free radical scavenging of the formulation was done to evaluate its cytotoxicity against RB cells [129].

### 6.2. Gold Nanoparticles (AuNPs)

**Figure 4:**
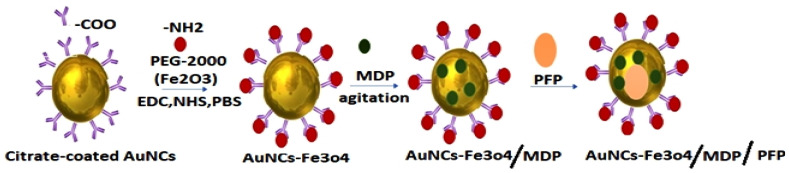
Conjugated (AuNCs-Fe_3_O_4_) nanoparticulate system modified to encapsulate the muramyl dipeptide (MDP) and perfluoropentane (PFP) to develop AuNCs-Fe_3_O_4_/MDP/PF.

AuNPs have been recruited for therapeutic efficacy owing to their large surface areas, enabling the adsorption of several functional molecules on their surfaces. However, they have limited applications owing to their toxicity [130,131,132]. To address these concerns, Kalmodia et al. (2017) developed green synthesis methodology for the synthesis of gold nanoparticles (GNPs) using extracts of *Vitus vinifera* [133]. The GNPs synthesized were biocompatible and noncytotoxic. These NPs were found to be involved in the mechanistic approach of knocking out human double minute 2 (HDM2) functional protein cells. HDM2 is a cancer target, as it inhibits p53 tumor suppressor activity. These HDM2 cells are knocked down due to their overexpression in retinoblastoma. Chen et al. (2020) also developed rosiglitazone-incorporated AuNPs in treating human RB [108]. The investigation of the anticancer activity, proliferation, and apoptosis of retinoblastoma cells were determined via flow cytometry. Furthermore, phosphoinositide 3-kinase inhibitor (PI3K inhibitors) were used to explore whether rosiglitazone AuNPs play a regulatory role through the PI3K/Akt pathway. The results concluded that the synthesized formulation significantly reduced the proliferation and antitumor activity, as well as apoptosis, of retinoblastoma cells as compared to the untreated controls. Wang et al., in 2020, developed multi-functionalized NPs for low-intensity focused ultrasound-assisted imaging for the synergistic treatment, as well as diagnosis, of RB [79]. This novel formulation was synthesized via the conjugation of magnetic hollow mesoporous gold nanoparticles (AuNCs) and iron oxide NPs (Fe_3_O_4)_. The prepared conjugated (AuNCs-Fe_3_O_4_) nanoparticulate system was then modified to encapsulate the muramyl dipeptide (MDP) and perfluoropentane (PFP) to develop AuNCs–Fe_3_O_4_/MDP/PFP, as shown in Figure 4. The synthesized nanoparticulate conjugate was successfully characterized through TEM, FTIR, the loading efficiency, release, in-vitro cytotoxicity, apoptosis, magnetic targeting ability, in-vivo therapy, and biocompatibility. The most important characterization of multifunctional NPs includes the systematic biosafety evaluation. The results concluded that multifunctional magnetic AuNCs-Fe_3_O_4_/MDP/PFP nanocarriers resulted in enhanced photoacoustic imaging, as well as magnetic resonance. After the directed entry of these nanocarriers, they get accumulated via a magnetic field in the tumors, and MDP was released under phase transition and irradiation. MDP, after its release, promoted the maturation and activation of the dendritic cells for an enhanced recognition capability for clearing tumor cells and showing great potential as an advanced therapeutic output against RB [79]. Scientists have developed metallic nanoparticles (silver and gold) to mitigate RB malignancy, but metallic nanoparticles have severe toxicity issues. In our opinion, the green synthesis of metallic nanoparticles can be encouraged to achieve anticancer activity with no toxicity.

## 7. Conclusions, Challenges and Perspectives

Compared with conventional anticancer therapies, NP-mediated anticancer drug delivery leads to high therapeutic efficacy, less toxicity, targeted binding with the ligand, and site-specific delivery, resulting in cytotoxicity management and cost-effectiveness. The barriers in the treatment of RB and the killing of healthy cells have been minimized via using biocompatible polymers such as ligands and green synthesis-based metallic NPs, as well as bioactive nontoxic herbal flavonoid constituent-based lipid nanoparticles. Emerging trends of multi-functionalization and biocompatible ligands in anticancer therapy and diagnosis are opening a new era in overcoming the barriers of conventional therapies via strategically improving the treatment and diagnosis of RB. Experimental studies are designed to establish cell/tissue-specific nanosystems to meet the challenging requirements of intraocular chemotherapy and diagnostics, with the revolutionary advancement of nanomedicine in cancer diagnosis. The design concept criteria and our current understanding of the general drug delivery and imaging of the eyes were outlined in this study. To achieve the ultimate objective of developing “smart nanosystems” against potent lethal intraocular tumors, combinatory strategies should be designed to suit different design parameters. The challenges in RB treatment involve distinct and complicated anatomical and physiological barriers in the diseased area of the eye, leading to drug spillage and non-targeted delivery, causing therapeutic inadequacy. However, the use of multi-functionalized ligand-based nanocarriers helps in maintaining the therapeutic efficacy via targeted actions.

## Figures and Tables

**Table 1:** Summary of several nanostructures in the diagnosis of retinoblastoma (RB). [NPs: nanoparticles, QDs: quantum dots, MT: metallothionein, PT-OCT: photothermal optical coherence tomography, and Au: gold].

Nanostructure	Key Feature	References
**Gold NPs**	Due to selective light absorption by the administered gold NPs, photoacoustic image contrast from the tumor regions was improved.	[73]
**Gold nanoclusters**	The signal enhancement by >500 gold atoms in each nanocluster enabled laser ablation (LA) coupled to inductively coupled plasma—mass spectrometry (ICP-MS) to image the antigens (MT 1/2 and MT 3) using a laser spot size as small as 4 μm.	[74]
**Gold nanorods**	The effectiveness of PT-OCT, along with Au nanorods, to picture the distribution in the mouse retina of both endogenous and exogenous absorbers.	[75]
**Magnetic NPs**	In magnetic resonance imaging (MRI) studies, the nanoparticles displayed perfect negative contrast and demonstrated their biocompatibility without cytotoxicity (5–100-μg/mL Fe_3_O_4_ NPs) to both regular and cancer cells.	[76]
**Quantum dots**	The preservation of QDs in the cryogenically injured corneal endothelium mouse model eyes was from 3 to 48 h post-cell injection on the posterior surface but not in the non- injured stable control eyes.	[77]
**Carbon nanomaterials**	The quantitative identification of the DNA methylation ratios was only calculated by methylated 5’-cytosine-phosphoguanosine (CpG) repeat oligonucleotides (60 mers) with various methylation ratios by carbon nanofilm electrodes.	[78]
**Multi-functional NPs**	In vivo and in vitro, mesoporous Au nanocages (AuNCs) combined with Fe_3_O_4_ nanoparticles improved photoacoustic (PA), ultrasound (US), and magnetic resonance (MR) imaging, which was beneficial for diagnosis and efficacy monitoring.	[79]

**Table 2:** Summary of several nanocarriers in the treatment of RB.

Nanocarrier	Key Feature	References
**Melaphalan NPs**	The double-emulsion method was utilized to reduce melphalan spilling during the fabrication process and resulting in targeted delivery	[102]
**Galactose NPs**	In RB, sugar moieties in the form of lectins are highly overexpressed as compared to healthy cells.Therefore, galactose is a mean of targeting for achieving efficacious results.	[103]
**Hyaluronic acid NPs**	Nonviral polymeric gene DNA complex-based nanomedicines were coated electrostatically with hyaluronic acid (HA) for providing an anionic hydrophilic coating for improved intravitreal mobility.	[104]
**Folic acid NPs**	Chitosan NPs (CNPs)and loaded doxorubicin (DOX) were synthesized and conjugated with folic acid for targeted delivery against RB.	[105]
**LipidNPs**	Switchable lipid nanoparticles (LNPs) were synthesized for the codelivery of melphalan and miR-181, having 93% encapsulation efficiency against RB.	[106]
**SilverNPs**	Silver nanoparticles (AgNPs) via rapid methodology from natural sources of brown seaweed *Turbinariaornate* and its cytotoxic efficacy were determined against RB cells.	[107]
**Gold NPs**	In vivo and in vitro, mesoporous Aunanocages (AuNCs) combined with Fe_3_O_4_NPs improved photoacoustic, ultrasound, and magnetic resonance imaging, which was beneficial for diagnosis and therapy.	[108]

## Data Availability

Not applicable.

## Abbreviations

NPs	Nanoparticles
AgNPs	Silver nanoparticles
CD44	Cluster of differentiation 44
CNPs	Chitosan NPs
DCM	Dichloromethane
DOX	Doxorubicin
EDTA	Ethylenediamine tetra-acetic acid
FA	Folic acid
Fe_3_O_4_	Iron oxide
GNPs	Gold nanoparticles
LNPs	lipid nanoparticles
MDP	Muramyl dipeptide
PFP	Per fluoro pentane
PLGA	Poly-d,l-lactic-co-glycolic acid
RB	Retinoblastoma
